# Translational Research in Cancer Screening: Long-Term Population-Action Bridges to Diffuse Adherence

**DOI:** 10.3390/ijerph18157883

**Published:** 2021-07-26

**Authors:** Lea Hagoel, Gad Rennert, Efrat Neter

**Affiliations:** 1 Department of Community Medicine and Epidemiology, Carmel Medical Center and Faculty of Medicine, Technion, Haifa 3436212, Israel; mdlea@technion.ac.il (L.H.); rennert@clalit.org.il (G.R.); 2 Department of Behavioral Sciences, Ruppin Academic Center, Emeq Hefer 4025000, Israel

**Keywords:** cancer early detection, cancer screening, communication, interdisciplinary, public health, translational medical science

## Abstract

The population-level implementation of innovative, evidence-based medical recommendations for adopting health-behaviors depends on the last link in the translation chain: the users. “User-friendly” medical interventions aimed at engaging users to adopt recommended health behaviors are best developed in a collaborative bio-medical and social sciences setting. In the 1990s, National Breast and Colorectal Cancer Early Detection Programs were launched at the Israeli Department of Community Medicine and Epidemiology. Operating under the largest HMO (Health Maintenance Organization) in Israel (“Clalit Health Services”), the department had direct access to HMO community primary-care clinics’ teams, insured members, and medical records. Academically affiliated, the department engaged in translational research. In a decades-long translational process, this multi-disciplinary unit led a series of interventions built upon basic and applied behavioral/social science phenomena such as framing, “Implementation Intentions,” and “Question-Behavior-Effect”. A heterogeneous team of disciplinary specialists created an integrated scientific environment. In order to enhance screening, the team focused on the establishment of a systematic mechanism actively inviting programs’ “users” (average-risk targeted individuals on the national level), and continuously applied social and health psychology concepts to study individuals’ perceptions, expectations, and needs related to cancer screening. The increase in adherence to screening recommendations was slow and incremental. A decrease in late-stage breast and colorectal cancer diagnoses was observed nationally, but participation was lower than expected. This paper positions screening adherence as a unique challenge and proposes new social and network avenues to enhance future participation.

## 1. Introduction

The translational research attempts to narrow the gap between discoveries or innovations and their dissemination to clinicians, patients, and individuals in the community. In the mid-1990’s, epidemiology experts concluded [1,2,3,4,5,6] that screening for the early detection of breast and colorectal cancers would be advantageous for individuals and target audiences. At the population level, higher rates of early-stage disease diagnosis were expected, and a higher likelihood of recovery and mortality reduction. Other medical specialists were becoming acquainted with this innovation, but for laypersons, receiving an invitation to test for a “problem” they did not present to their attending physician raised eyebrows, even concern. Diffusing the screening message and achieving participation in breast and colorectal cancer screening presented a challenge (illustrated in detail under “Stage 0”, pp. 5–7). From a purely medical-rationale point of view, this should have been an “easy” challenge to meet, in light of the known advantages of undergoing screening tests, but reality did not support this assumption. In cancer screening, this gap was and still is wide in many places [7], requiring an explanation. 

### 1.1. The Context of Translational Research

Interventions for enhancing health behaviors depend on the cooperation of the individuals addressed (the target population) and on the implementation of biomedical evidence-based interventions supported by scientific knowledge of human behavior. Collaboration between social and medical experts in transdisciplinary partnership is required. Discussing the dynamics between scientific disciplinary specialization in issues extending beyond any single disciplinary specialization, collaboration and translation were inevitable [8]. Founded upon this very idea [9], the Department of Community Medicine and Epidemiology (“the department”) illustrates such collaboration. 

#### 1.1.1. Multi-Disciplinary Organizational Structure 

Academic departments generally house staff members of a similar disciplinary background. “The department” was established within the Technion’s Faculty of Medicine, with a *heterogeneous team.* Various disciplinary experts, located together, shared an agenda and worked together in this organizational unit: epidemiologists, family physicians, biostatisticians, social scientists, molecular biologists, laboratory technicians, information technology experts, and administrative personnel, all under one roof with a holistic view of the individual [10]. A structurally heterogeneous group genuinely collaborated. Sharing an organizational affiliation provided informal opportunities to get acquainted, rooting for transdisciplinary work patterns. Each discipline represented was granted equal legitimacy and non-judgmental acceptance. *Lesson learned:* The contributions of all were respected based on merit and relevance, which cannot be taken for granted [11].

Spatial proximity strengthened social interaction patterns and mutual learning of “neighboring” scientific disciplines. A growing understanding of concepts facilitated the emergence of a common language, combining central terms from all involved disciplines. In a decades-old weekly departmental “Journal Club”, academic articles representing the active disciplinary fields were discussed. Short introductory presentations familiarized the heterogeneous audience with the relevant basic terminology allowing a common “fund” of constructs to develop. *Lesson learned:* This investment helped create a link between basic and applied clinical science [12]; social science discoveries became accessible to medical experts involved in interventions for adopting health behaviors. Falling “between scientific cracks” [12] was avoided. 

Promoting trust, the multi-disciplinary context encourages innovation [13]: “…trust operates as a multi-dimensional construct, and in the department it is a construct which characterizes teams, and even the entire organization” [9]. Disciplinary bridging, a central element in translational research, thrives through the development of a *collaborative culture*, minimizing the built-in conflict in heterogeneous scientific encounters. Team members took their specialties to a variety of projects with different collaborators in each. *Lesson learned:* Experts worked with different individuals simultaneously, furthering acquaintance, trust, and first-hand knowledge of the usefulness and relevance of other disciplinary backgrounds to their own professional challenges. 

Taking responsibility for the entire translational cycle was a collaborative action. Social scientists were responsible for introducing basic social scientific concepts, theories, and techniques. Physicians and epidemiologists conveyed information regarding medical and epidemiological aspects of screening: age criteria, frequency, medical risk, and meanings of specific terms. This meant that working the hypotheses, with regard to enhancing screening in the program’s target population, incorporated medical, epidemiological knowledge of the screening field and cancer etiology, as well as social scientific conceptualizations of perceptions of cancer, individuals’ perceptions about cancer early detection, mechanisms of coping, fear, changing behaviors, adopting new ones, and many more. The program no longer used a “purely medical” nor a “purely social scientific” perspective, as the relevant concepts from both fields of knowledge were fused together to create a relevant population-level intervention (see Section 3.1, Professional Assumptions). 

The process of multi-disciplinary bridging was expressed in the situational distribution of language functions [14], identified in intra-departmental communications [9]: “a variety of languages are heard: biological, laboratory, sociological, epidemiological, computers, bureaucratic-administrative and statistics—are used interchangeably as needed… Listening to one another, individuals can tell when the limits of their knowledge are reached, and new constructs appear, with little hesitation, they can then extend those limits through dialogue” [9]. *Lesson learned:* multi-disciplinary bridging requires acceptance. 

#### 1.1.2. A Dual-Organizational Affiliation: Academia and Medical Service Combined

With this professional makeup, the department relied on its *dual affiliation*: academic, with the Faculty of Medicine, and clinical, that of “Clalit Health Services” (CHS), Israel’s largest HMO (not-for-profit) healthcare provider. 

At the time, CHS-insured members comprised about half of the country’s population. It offered a centralized healthcare delivery system, with over 1000 primary-care neighborhood clinics in the country, providing services for insured members of all ages and their families. CHS insured members attend primary healthcare clinics located in their residential neighborhoods over decades. The local medical teams provide care based on long-term acquaintance. The department became CHS “Clalit Cancer Control Center”. 

The departmental multi-disciplinary team acquired direct access to community primary-care clinics, collaborating with the local medical teams, approaching clinics’ insured members, as well as having access to population-level medical records. Once the Cancer Control Center worked with the “field”, the development and implementation of a full translation cycle became possible: CHS medical teams in primary care neighborhood clinics across the country supported the central cancer screening programs initiated by the department.

Motivation and comfort of primary care teams in conveying preventive medicine messages are essential. The screening message is not always suitable for the clinical situation. Patients asking for the physician’s assistance present a complaint. Immersed in their problem, they may not be able to pay attention to a cancer screening recommendation; they may even be resistant to such a message. The close, often daily, work relations of the center’s team with the neighborhood clinic teams were a source of support. This also ensured the fidelity of the screening recommendations and the precise implementation of screening criteria for the average-risk target population. 

## 2. Screening for the Early Detection of Cancer: Programs and Interventions along Translation Stages 

In the early 1990s, epidemiologists in the department launched *a population-level cancer screening field experiment*. Based on these experiments [15,16] (see below, stage 1–2, pp. 7–8), the department initiated a nation-wide breast cancer screening program (using mammography) and an initially regional colorectal cancer screening program (using the fecal occult blood test (FOBT)), later becoming nation-wide: a provider-initiated, direct, mailed contact/invitation to everyone in the defined target population, regardless of clinic visits. Using “case-finding” would not reach everyone in this population, depriving them of equal access to the program. Centralized invitations were sent to the homes of all insured members in the target population: 50–74-year-old asymptomatic, average-risk women and men living in the community. This *continuously* ongoing activity covered the field research-intervention-evaluation-feedback-intervention refinement-by-lessons-learned cycle, on to the next intervention, over nearly thirty years: disseminating information, sending invitations, providing responses to concerned invitees, and returning test results to attending physicians, among others. “Interventions” refer to focused projects, specifically planned, carried out, and evaluated within the ongoing programs. 

In 2005, the Israeli Ministry of Health issued a formal policy recommendation to screen average-risk asymptomatic individuals for breast and colorectal cancers, free of charge, under the Israeli National Health Insurance law [17]. The anatomy of the two screening programs is presented in Appendix A. 

### Translational Research in Cancer Control: “NIH Stage Model” Illustrated

The NIH “stage model” conceptualizes intervention development [12] starting from basic scientific research and proceeding to dissemination research, with innovations adopted by its target audience(s). The stage model phases in intervention development are: basic science addressing fundamental constructs, such as health perceptions, prevention, biological processes (stage 0); intervention creation and modification (stage I); intervention testing (stage II); efficacy testing in a community setting (stage III) focusing on internal validity; effectiveness testing in a community setting (stage IV) focusing on external validity; implementation and dissemination (stage V), focusing on incorporating interventions into community settings. 

Adaptable to any scientific discipline, bio-medical or social, *the model is applied here to the behavioral sciences in the public health domain of cancer control*. Medical and behavioral aspects of clinical science were combined to develop population-level implementable interventions, bridging the science-to-service gap [12]. Medically initiated, the translational phases were infused with basic and applied social science know-how regarding encouraging individuals in the target population to participate (illustrated in Table 1); as adherence to cancer screening rose slowly, the weight of the social sciences increased along with the translational processes. Mixed methods were used throughout the stages, and interventions addressed self-regulation, stress reactivity, stress resilience, interpersonal, and social processes [18]. The scientific disciplines involved in the translational process included epidemiology, public health, sociology and psychology of health, and bio-statistics, supported by IT, administrative and laboratory teams. 

## 3. Intervention Cycle along NIH Stage Model

### 3.1. Stage 0 (Basic Science): Translating the Medical Message into Laypersons Terms

**Professional assumptions.** The initial program development stage involved a two-way apprenticeship: social scientists were introduced by epidemiologists to the cancer screening knowledge field, and basic assumptions of cancer screening were questioned by the social scientists. A qualitative data collection emerged, with non-judgmental observations and meticulous distinctions between evidence and interpretation of the (medical) screening rationale. Basic assumptions and terms questioned included “early detection”, “positive result”, “a screening indication”, as opposed to a diagnostic indication, “non-adherence”, “screening criteria”, and the handling of biological materials.

Medical terminology was partly recognizable in the physicians’ spoken language: Latin words were easily identifiable as medical jargon, but occasionally everyday words had a unique medical meaning attached to them. A layperson could not know they did not understand the physician’s intent. For example, “early” often refers to the time axis. Laypersons tended to assume that if “right after I discover a lump in my breast, I will go see my doctor, immediately, the same morning”—they will have succeeded in achieving “early detection”. However, physicians referred to the medical meaning, “early in the natural history of the disease”, in terms of its developmental stages. The use of “positive” as medical jargon, which does not carry the regular meaning of the term (the opposite of “negative” in judgmental terms), was confusing too. A person would ask: “I got a positive test (FOBT) result... Is this good or bad?”

The concept of screening was not easily acceptable to the public, and its rationale needed elaboration. A cancer screening recommendation differed from medical recommendations in clinical encounters following symptoms’ presentation: this was a novel socio-medical situation. The screening rationale was perplexing: complaining about symptoms and follow-up testing was the familiar medical care routine. A test referral initiated by the healthcare provider required adjustment by invitation receivers. The recommended pre-set frequency of testing (annually for colorectal cancer (CRC) early detection, bi-annually for breast cancer), puzzled individuals (“I performed the test last year”, they said when receiving a renewed test invitation); so did the definition of the programs’ target population: “why should everyone my age undergo this test?” 

An initial *lesson* was that screening criteria implicit in the message needed to be explicated, too. Undergoing medical tests while being asymptomatic was new, as was the difference between a diagnostic and a screening indication. This confusion was intensified by the medical procedure itself being identical in both cases. No *visible* signs pointed to an indication for test performance: a mammogram was a mammogram, whether the woman undergoing it was symptomatic or not. 

The gap between lay perceptions and medical definitions became clearer: women’s self-perceptions did not include the medical label of non-adherence assigned to them in breast cancer screening. They explained their non-attendance of pre-scheduled mammography appointments by reporting not having performed the test “yet”; *lesson learned:* none viewed themselves as “non-adherent” [15]. 

A referral to FOBT carried additional obstacles. The medical assumption was that a “take home” test performed in one’s privacy, on one’s own schedule, would be more likely to be adopted than testing in a designated medical clinic. However, though a paper-sheet that emerges on the physician’s printer, referring a person to undergo FOBT is identical to any blood-test or X-ray referral, FOBT had no medical facility address. *Lesson learned:* a shift in mindset was required to perform this screening test: from being passive in the diagnostic situation to being active, taking complete charge (including handling one’s biological materials). Reactions included: “I’ve got a test kit at home, have read the instructions, left the envelope closed, until I am ready.” Until pointed out by the social scientist, the medical team ignored the different expectations from the receiver in the case of a “regular” medical procedure versus the take-home situation. The difficulties were addressed in a variety of supportive interventions (stages 1 and 4 below).

*Lesson learned*: “The gap in knowledge of the principles of CRC screening that separates doctors and individuals invited for screening consists of language, concepts, perceptions, and assumptions unique to each of these two groups” [19]. 

**Laypersons’ concerns and motivations.** Understanding individuals’ concerns could assist invitees to participate in screening. Uncertainty, a long-term sense of vulnerability, fear and anger, feeling ignored by the healthcare provider, and self-perceptions stood out. *Lesson learned*: there was a need to reframe the healthcare situation, from “I have a medical problem, therefore I’ll undergo tests” to “I’m (healthy, yet) at risk to develop cancer, therefore I need to undergo tests before symptoms/medical problems develop”.

Screening recommendations were based on aggregate epidemiological data. Invitees asked: “What does it mean that I am at risk for developing cancer?” Even with the information provided, ethical criteria observed, and individual autonomy recognized, suffering was bound to result. Population-level benefits co-exist with the price “paid” at the individual level. *Lesson learned*: the team realized that being asked to undergo periodical screening tests continuously for decades reminded the patients of their being *at-risk to develop cancer*, impaired one’s quality of life. Assigning the at-risk label alters ones socio-medical status [20]: on the Healthy-Sick axis, their “healthy” status is questioned without defining them as “sick”, leaving them in limbo, with a sense of helplessness, hence, existential suffering, as individuals “ponder their lives… life’s purpose… achievements, relationships” [21]. “I’m anxious about every test (FOBT)… even the preventive ones…waiting for the results I constantly think about this…every year I debate … whether I should skip the test … it’s too stressful!” [21]. Rarely is this limbo considered a “screening barrier”. 

Another *lesson learned* was that fear may be defused over time as the innovation becomes familiar. Callers to the program’s office would ask: “What do you know about my family that’s behind this screening-test invitation?” Or say “I refuse to hear the news; thinking about a bad result raises fears”. *Lesson learned*: other respondents understood that any result of a screening test is “good”, in the sense that a disease diagnosis would, likely, be in its early, more treatable stages.

A fresh angle on enhancing cancer screening was obtained by focusing on individuals who did accept the invitation. Using an approach derived from Personal Construct Psychology [22], women waiting for their pre-scheduled mammogram in a radiology clinic provide their reasons to test. Their self-definition included holding a positive worldview, loving life and people, being honest and fair, and willing to help others. They accepted responsibility for themselves and for family members and “brought along” their fears to the testing situation, refusing to give in to them. These were friends who scheduled bi-annual mammograms on the same morning, just as their weekly coffee get-together, supporting each other through the procedure. What intervention could cause women to adopt such a view on life? *Lesson learned*: having shared this finding with primary care medical teams; screening appointments were turned into a social event for rural women. Mammograms were scheduled for them on the same morning; riding together in a mini-van to an urban clinic for their appointments, they could later enjoy a visit to a nearby mall. 

### 3.2. Stages 1–2: Intervention Development and Modifications

Initial experiments preceded the establishment of the two programs. The one on mammography screening assessed women’s views and reactions to a pre-scheduled invitation [15]. Among participants, 16% had performed the test before the study, 45% thereafter. The second experiment assessed women;s and men’s responses to different FOBT invitation versions (with/without an information leaflet, with a kit, or with a kit-order card) [16]. FOBT testing increased following the invitation from 0.6% to 17.9%.

Participants in both surveys were interviewed over the telephone using similar questionnaires with more health-behavior questions to women invited for mammography. Adherence to mammography screening was identified as a marker for background variables, health behaviors, and cancer-related perceptual variables [23]. *Lesson learned*: groups identified were *self-screeners*, who adopted health behaviors more than *adherent* women; the latter adopted these behaviors more than *non-adherent* participants [24]. Two strategies were recommended for enhancing adherence to mammography screening: the direct (encouraging individuals to adopt a recommended behavior) and the indirect approach (encouraging the adoption of the health behavior cluster lifestyle).

Hebrew messages were sent to invite CHS-insured members for screening; Arabic, and Russian speakers asked to receive them in their own languages. Once translated messages were included, others complained, claiming they did not need a translation. *Lesson learned*: print all three linguistic versions on one sheet. 

### 3.3. Stages 1,3: Efficacy Testing

Stage 3 intervention development focused on the community setting and on improving the program “mechanics”. To assure fidelity in the screening messages, physicians, nurses, and administrators in the community primary-care clinics were provided with guidance. Ongoing program monitoring and quality control of IT, laboratory, and medical outcomes were carried out to tighten program procedures (see Appendix A). 

Continuous medical quality control was enabled by the departmental organizational affiliation with the HMO and was part of the programs’ regular activity. The mammography examination service was another quality indicator examined, and specifically, women’s satisfaction, associated with willingness to repeat the test. A random sample of 3295 women tested in 38 units nation-wide was drawn. Within 48 h of their mammography, they were asked to assess the examination process, discomfort, overall satisfaction, and intention to rescreen. *Lesson learned*: though satisfaction and intention to rescreen were high, the observable gap between intention and rescreening required further investigations of other types of barriers to regular, repeated mammography screening [25]. 

### 3.4. Stage 4: Effectiveness Trials

Over a decade into the CRC early detection program, two large-scale initiatives were undertaken. Intervention developers identified, in the literature, behavior change techniques that demonstrated efficacy in the laboratory and in small and mid-scale field interventions. These techniques were adapted to the population level, enabled by the access to (1) the mailing lists, (2) the phrasing of messages for insured members, (3) IT infrastructure, and (4) the HMO legal support. 

Implementation intentions (II) [26,27,28] focused on the screening planning, targeted interested insured members, and assisted them in self-regulation. Preliminary qualitative work identified obstacles in carrying out screening intentions (e.g., choosing the date (*when*); *where* to store the kit between samplings) [29]. Individuals who screened in the previous year were identified as holding positive intentions towards screening, and a “thin” II intervention was mailed to them: an illustrated leaflet containing an “*if–then*” condition with planning instructions: when, where, and how to perform the test (see Supplementary Materials). The departure from interpersonal contact and respondent-generated solutions for overcoming obstacles carried out in laboratory contexts, allowed upscaling the behavior change technique to the population level. In an effectiveness trial, 29,833 HMO insured members were randomly assigned to standard care or to an II experimental group receiving the designated leaflet. *Lesson learned*: the II technique was useful in increasing adherence to CRC screening in a mailed form rather than a face-to-face experimental situation. An inexpensive, effective method, it was recommended for public health [29].

The second effectiveness trial capitalized on an unconscious process whereby asking questions about future behavior increases the likelihood of performing this behavior (“question-behavior-effect” [30,31]). This technique was adopted to the population level by using a single text message (SMS) sent to insured members’ smartphones, worded as a statement or an interrogative CRC screening reminder. Following an FOBT invitation, reminders were sent to 50,000 randomized women and men in the program’s target population. *Lesson learned*: increased screening was modest; absolute numbers of participants, population-wise, suggested a clinically significant health promotion change [32].

Text message respondents to SMS interrogative reminders screened significantly more than non-respondents, six months, one, and two years following the reminders [33]. *Lesson learned*: interrogative SMS reminders reached previously uninvolved sectors in the CRC target population—men, sporadic-screeners, and “never-tested” before.

### 3.5. Stage 5: Dissemination

Dissemination work was carried out collaborating with national health policymakers and with international screening frameworks. Intervention developers became a “knowledge center” for approaching insured members to enhance adherence within CHS.

Following the International Cancer Screening Network (ICSN) meeting in 2008, a Screening Participation Rates Work Group was formed, with the CHS program represented. The workgroup assessed an international comparison of CRC screening participation rates across organized guaiac FOBT/FIT-based programs (15 programs in 12 countries), examining associated factors. Differences in organization, target populations, and indicator interpretation were highlighted [7]. *Outcome*: Concomitantly, The Israel National Program for Quality Indicators in Community Healthcare included cancer screening as an indicator [34]. This positioned cancer screening as a backbone of preventive medicine, tied to performance targets of neighborhood clinics and regional units. 

Each lesson learned had been incorporated into the program, and following the SMS interrogative reminders study publication, the program’s team was contacted by CHS headquarters for consultation on using QBE to enhance other health behaviors.

## 4. Conclusions

An organizational structure facilitated the development of transdisciplinary collaboration, yielding a productive translational-research environment. The Israeli population became familiar with cancer screening while adherence to screening invitations was enhanced. 

### 4.1. Strengths

Increased screening rates, message dissemination: from 16% and 0.06% in breast and CRC screening [15,16], in 1995, respectively, to 70.5%, and 63.3% in breast and CRC screening, respectively, in 2017 [34]. International CRC participation data of organized screening programs ranged between 7% and 67.7% ([7] Table 3).

Disease stage at diagnosis: in 2015, 64% of all new breast cancer patients were diagnosed at an early disease stage, as compared to 58% in 2005 [35]. Patients diagnosed with metastatic breast cancer were 2.6% in 2015 compared to 3.6% in 2005. In CRC, patients diagnosed at an early stage were 29.6% in 2016 compared to 19.9% in 2000; patients diagnosed at a metastatic stage were 9.3% in 2016 compared to 14.6% in 2000, a 36% decrease [36]. This impact was attained by combining theory [37] and target audience inputs. The fusion of medical and social theories and practices was propelled primarily by organizational-structural features of transdisciplinary collaboration.

The continuous, longitudinal effort to enhance adherence on the population level included applying lessons learned in a multi-disciplinary context and integrating biomedical and social scientific knowledge. The existence of a program afforded this continuous process of refinements and realizations of “lessons learned”. The public was availed not just coverage for screening, but the responsibility for screening was shared between the public and the interdisciplinary team, the latter ethically committed to health equity. The comprehensive invitation mechanism, which included everyone in the target population, made sure that equal screening opportunity was offered to all eligible HMO insured members. The quality control processes, new initiatives of behavior change techniques, and close collaboration with neighborhood clinics and regional units incrementally prodded more screening participation. There are many “boxes” between a design of a screening program, its implementation (which involves multiple collaborators), and the health outcomes.

### 4.2. Limitations

First, a rounded understanding of the medical rationale of screening by the target audience is still to be reached [38]. Rather than screening in the pre-specified frequency, sporadic screening is observed. Sometimes, the required follow-up procedures in cases of a positive screening test are avoided, often because of misunderstandings regarding the procedures, delaying diagnosis. The screening rationale is not accepted, tabula rasa; to be adopted, it needs to replace (if possible) existing lay perceptions and interpretations. Second, even motivated individuals, who have positive perceptions of screening tests and see their potential benefits, often have difficulty realizing their intentions to undergo a screening test. Accordingly, self-management interventions to assist the target audience in realizing their intentions need to be included in screening services. Third, some of the important variables affecting the adoption of screening and of other health behaviors (e.g., social class, perceived health) are not amenable to interventions, as are others. Fourth, there is no direct evidence connecting the string of interventions to population/country level screening participation, especially as the health outcomes (screening rates, cancer stage at diagnosis) are multi-factorial and were affected by many individuals and organizations along the service chain. Still, accumulated evidence in our randomized experimental interventions provides sample-level evidence (e.g., n = 50,000). In some cases, the effect lasted for two years [33]. 

Today the public is more knowledgeable than in the early 1990s, at the beginning of our work. The future of enhancing adherence to screening will require new avenues to be systematically explored on the group and individual levels. Partnerships based on social processes and group support for individuals interested in sharing resources to further the health of a community, a neighborhood, or a friendship network. For example, mutual support, as illustrated by women scheduling their mammography on the same morning or by individuals thinking of their peers as they decide to undergo a screening test, may be a successful next social intervention. 

Finally, conducting population screening is a highly dynamic and complex process, ranging from policymaking through performance by target audiences to resource allocation. This requires repeated scrutiny of the overall rationale for routine cancer screening of millions of people and continuous assessment of all related elements (e.g., adherence rate, positivity rate, colonoscopy rate, disease detection/mortality rates, costs).

## Figures and Tables

**Table 1:** The translational process: research—intervention cycle along NIH Stage Model.

Stage	Breast/Colorectal Cancer (CRC) Screening Programs: Research-Based Efforts	Evidence-Based Intervention Upgrade and Refinement by Lessons Learned
0 Basic Science pp. 5–7 tighten program procedures	Introducing the cancer screening idea: Identifying language gaps between public health professionals and laypersons. Methods: qualitative	Training medical teams in primary care clinics on approaching insured members
1–2 Intervention Development and Modification p. 7	Organized initiated invitations: Feasibility studies psycho-social determinants of health behaviors. Methods: quantitative	Establishment of screening programs for the early detection of breast and colorectal cancers (interventions’ umbrella); Adjustment to the population sub-groups unique needs
1,3 Efficacy Testing pp. 7–8	Expanding/refining procedures: Assuring fidelity, continuous quality control, IT support Methods: qualitative, quantitative	Identify gaps between an individual’s intention and actual behavior (rescreening). Continued training medical teams in primary care clinics on approaching insured members
4 Effectiveness Trials * p. 8	Introducing behavior-change techniques: Implementation intentions and interrogative reminders. Methods: quantitative	Adopted: new instructions leaflet (Supplementary Materials) incorporated: Health interventions
5 Dissemination * pp. 8–9	International collaboration: serving as a knowledge center within the HMO	

* Breast cancer screening rates were rising faster than CRC screening rates, further modification focused on adherence to CRC.

## Data Availability

Not applicable.

## Supplementary Materials

The following are available online at https://www.mdpi.com/article/10.3390/ijerph18157883/s1. Screening invitation and instructions.

## Appendix A

Anatomy of two screening programs: Breast and Colorectal Cancer Early Detection.

The programs worked according to similar principles, with separate administrative offices:

- IT personnel set up CHS-insured member databases, including their medical records and the software for each program, in line with the medical definition of the target population.
- Invitations to undergo the recommended screening tests, free of charge, were worded (variations were empirically compared as to their effectiveness in generating adherence). Timing and frequency of mailings are beyond the scope of this anatomy.
- Invitation for mammography screening included a pre-scheduled appointment with the clinic’s telephone number for women who wished to alter the date/time (this method was chosen following an empirical investigation by the departmental team).
- Invitations for undergoing an FOBT, a take-home test, used a two-step approach (this method was chosen following an empirical investigation by the departmental team): a kit-order card was attached to the invitation letter. Receivers of the letter who wished to undergo the test could mail the card back or call the office and order a kit to be mailed to them.
- The test kit includes an information leaflet about colorectal cancer and its early detection, instructions for using the kit to perform this take-home test, and a medical questionnaire, which, in positive cases, is communicated to the attending physician with the test result. These materials were prepared in collaboration by team members, tested empirically, and revised several times using social-psychological conceptualizations for assisting individuals to move from agreeing to test to actually performing it.
- PDF files of the invitation letters and data files with addressees’ contact information were forwarded to a printing house, which combined, printed, and mailed the invitations.
- For individuals who chose to undergo the tests, the CHS database was updated with all medical details: for mammography, the clinics would forward results (to the woman tested, and to the physician, where follow-up would be initiated according to the test results), and the FOBT kits were processed at the departmental laboratory, with technicians recording results directly into the computerized database.
- Medical monitoring and follow-up through treatment in positive cases constantly accompanied these under the same organizational roof, but this report is concerned only with the socio-behavioral aspects of interventions to enhance screening.
- Individuals’ test results are routinely forwarded to their primary care physician. Mammographic results are provided by the clinic, positive results of FOBT were sent to the attending physician by the program’s administration in three ways: regular mail, email, and fax, along with a letter recommending inviting the individual and referring them to undergo a colonoscopy.
- The programs are very concerned with the, still relatively large, part of the target population who do not respond to the invitations. This is less the case with invitations for mammography, but with FOBT, there are elaborate mechanisms embedded in the routine activity of the program, which includes various forms of reminders and invitation letters for those who just turned 50 or who did not respond for a few years.
