# Supplementary material for: Translational Research in Cancer Screening: Long-Term Population-Action Bridges to Diffuse Adherence
Source: Int J Environ Res Public Health. 2021 Jul 26;18(15):7883. doi: 10.3390/ijerph18157883 (PMC8345519; doi:10.3390/ijerph18157883)
Supplement: Supplementary file 1 [file ijerph-18-07883-s001.zip › ijerph-1275899-supplementary.pdf]

National Cancer Control Center
The program for the early detection of colorectal cancer

Hello,

It is time again for your annual FOBT.

What helps the most to perform the test is to set for yourself ahead of time – ***When, how, and where*** to do this.

If you already have the kit, ***then*** take advantage and ***use*** it!

We expect your kit soon!

Good luck!

Prof. Gad Rennert
Center Director

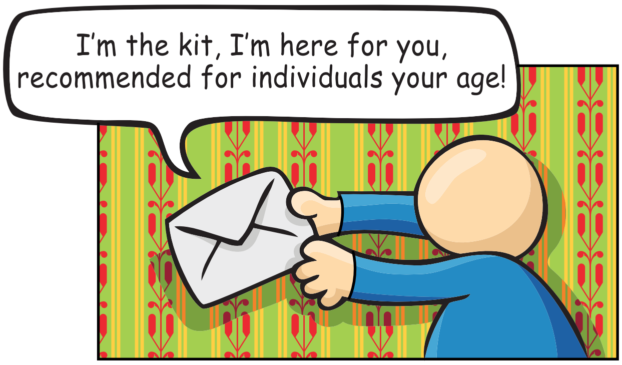

Fecal Occult Blood Test - *how to?*

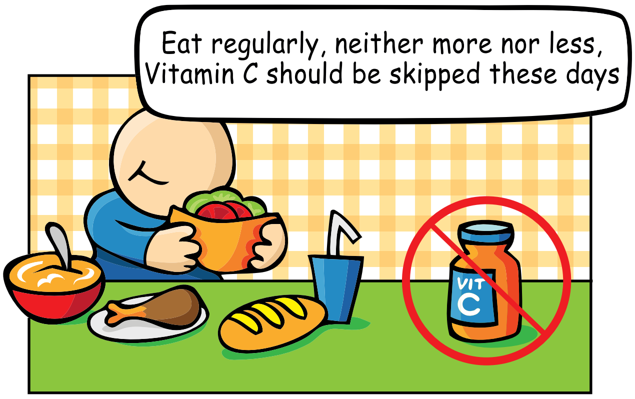

When would you like to begin?

A date needs to be set, for example, next Monday.

Please write it down here for yourself:

On (day) ___________, date _____________, I'll start the test (during the three preceeding days, avoid vitamin C supplements).

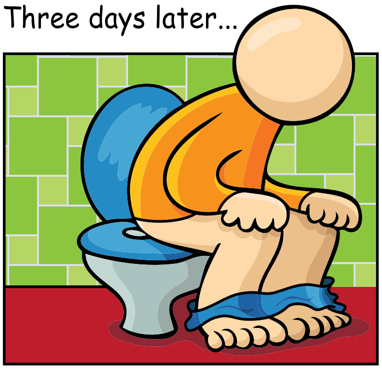

How:

Taking the the kit to the toilet, before each of the next 3 BM's (bowel movements) –

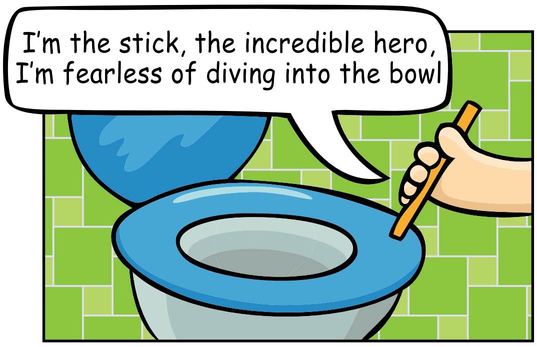

- Using the stick, take a tiny sample of feces onto one window in the first flap.
- With the same stick, repeat this in ***another part*** of the feces and place it in the ***other window*** in the same flap.
- Leave to dry for an hour and close the flap.

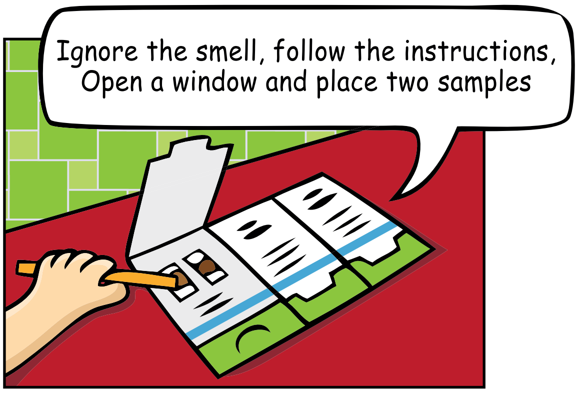

A bit smelly? It's not that bad.
Please repeat twice more.

Repeat this in the next flaps for the second and third BMs, and send the kit in the pre-stamped envelope.

Where (can I store the kit during the test days?)

In the privacy of your home, in the bathroom (possibly in an old box):

- On a shelf
- On the windowsill (careful, do not let it fall out) or in another suitable place.

It is important to let others in the house know, so they do not throw away the kit accidentally.
You can plan and note: where is it best to keep the kit in ***your*** bathroom?

____

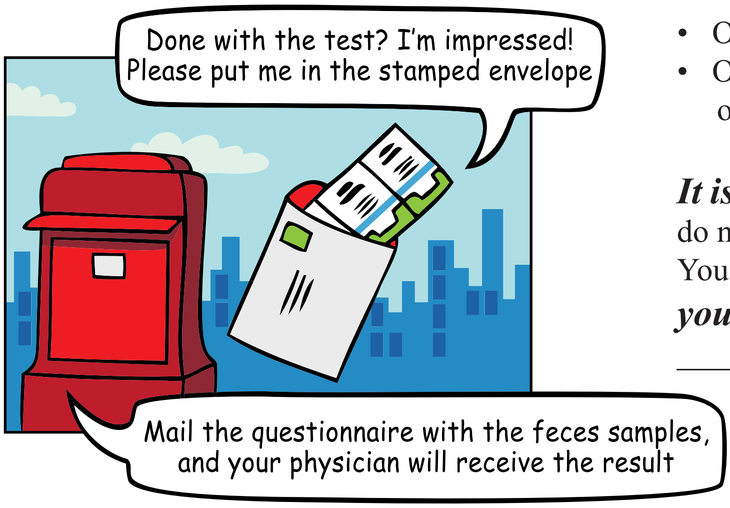

© Illustrations: Tali Ore Shalev
